# [NiFe]-hydrogenases are constitutively expressed in an enriched *Methanobacterium* sp. population during electromethanogenesis

**DOI:** 10.1371/journal.pone.0215029

**Published:** 2019-04-11

**Authors:** Elisabet Perona-Vico, Ramiro Blasco-Gómez, Jesús Colprim, Sebastià Puig, Lluis Bañeras

**Affiliations:** 1 Molecular Microbial Ecology Group, Institute of Aquatic Ecology, University of Girona, Girona, Spain; 2 LEQUiA, Institute of the Environment, University of Girona, Girona, Spain; Universidade Nova de Lisboa Instituto de Tecnologia Quimica e Biologica, PORTUGAL

## Abstract

Electromethanogenesis is the bioreduction of carbon dioxide (CO_2_) to methane (CH_4_) utilizing an electrode as electron donor. Some studies have reported the active participation of *Methanobacterium* sp. in electron capturing, although no conclusive results are available. In this study, we aimed at determining short-time changes in the expression levels of [NiFe]-hydrogenases (Eha, Ehb and Mvh), heterodisulfide reductase (Hdr), coenzyme F_420_-reducing [NiFe]-hydrogenase (Frh), and hydrogenase maturation protein (HypD), according to the electron flow in independently connected carbon cloth cathodes poised at– 800 mV *vs*. standard hydrogen electrode (SHE). Amplicon massive sequencing of cathode biofilm confirmed the presence of an enriched *Methanobacterium* sp. population (>70% of sequence reads), which remained in an active state (78% of cDNA reads), tagging this archaeon as the main methane producer in the system. Quantitative RT-PCR determinations of *ehaB*, *ehbL*, *mvhA*, *hdrA*, *frhA*, and *hypD* genes resulted in only slight (up to 1.5 fold) changes for four out of six genes analyzed when cells were exposed to open (disconnected) or closed (connected) electric circuit events. The presented results suggested that suspected mechanisms for electron capturing were not regulated at the transcriptional level in *Methanobacterium* sp. for short time exposures of the cells to connected-disconnected circuits. Additional tests are needed in order to confirm proteins that participate in electron capturing in *Methanobacterium* sp.

## Introduction

The term electromethanogenesis was first coined by Cheng and co-workers to indicate the reduction of carbon dioxide to methane mediated by *Methanobacterium palustre* using an electrode as electron donor [1]. Indeed, it was suggested that methane was directly produced from the electrical current as the sole source of energy and reducing equivalents. However, this may not be the general rule for electromethanogenesis since hydrogen-mediated CO_2_ reduction has also been observed in bioelectrochemical systems (BES), suggesting that the two processes may coexist [2,3].

Recently, researchers have focused on the detection of proteins likely involved in electron transfer mechanisms in order to elucidate biological mechanisms for the electrode-to-cell or cell-to-cell electron flow [4,5]. The participation in electron uptake of the heterodisulfide reductase supercomplex (Hdr-SC) and, also, formate dehydrogenase (Fdh) has been confirmed in the methanogenic archaeon *Methanococcus maripaludis*. Although the two enzymes are initially located in the cytoplasm, they can be exported to the outside of the cell coming in close contact with the electrode in which electron harvesting would occur [4,6].

Soluble hydrogenases, ferredoxins, formate dehydrogenase and cytochromes have been suggested to actively participate in initial steps of microbial electrosynthesis in BES containing biofilms enriched with *Acetobacterium* sp. and *Desulfovibrio* sp. [7]. Hydrogenases catalyze the reversible reduction of ferredoxin with hydrogen (H_2_) driven by a proton or sodium ion motive force (Fd_ox_ + H_2_ +ΔμH^+^/Na^+^ ⟺ Fd_red_^2-^ + 2H^+^) and are ubiquitously distributed among methanogenic archaea [8]. Hydrogen is used by hydrogenotrophic methanogens as the primary energy source for catabolism [8], but also fuels CO_2_ fixation during anabolism [9], and different anaplerotic functions [10], being the major energy source.

Different [NiFe]-hydrogenase subtypes are found in methanogens [8], and are likely to participate in energy conversion. Specifically, *Methanobacterium* sp. contains [NiFe] group 3, subgroups 3a (F_420_-coupled) and 3c (heterodisulfide reductase-linked) and 4, subgroups 4h (Eha) and 4i (Ehb) [11].

Eha and Ehb are the only enzyme complexes of the methanogenic electron transport chain to have membrane integral subunits [12]. Operons (ehaA-T and ehbA-Q) encode for several integral membrane proteins, hydrophilic subunits, two polyferredoxin subunits, and [NiFe] small and large subunits [12]. Ehb is specifically linked to CO_2_ fixation providing anabolic electrons for carbon assimilation [9] and, it is highly expressed in comparison to Eha (directly involved in the methanogenic pathway), at least in *M*. *thermoautotrophicus* [12].

Heterodisulfide reductase complex (Hdr) involves the participation of the mentioned cytoplasmic [NiFe]-hydrogenases and two dehydrogenases, formyl-methanofuran dehydrogenase (FwdABD) and formate dehydrogenase (FdhAB). The complex is involved in the methanogenic pathway from CO_2_ where H_2_ or formate donates electrons to it via Hdr-associated hydrogenase (Mvh) or formate dehydrogenase (Fdh). Also, coenzyme F_420_ (Frh) participates in the pathway as electron donor [8,13].

In addition, a series of cytoplasmic proteins (known as expression formation proteins HypA-F) participate in the maturation of hydrogenases [8]. One of these proteins, HypD, is highly conserved and essential for the maturation of hydrogenases. HypD contributes to the insertion of the Fe(CN)_2_(CO) moiety in the correct oxidation state into the active site of different [NiFe]-hydrogenases [14]. Due to this tight relationship, HypD is a good candidate to measure the expression of different soluble [NiFe]-hydrogenases.

Increasing the current knowledge on the mechanisms involved in electrode-to-cell electron transfer at the molecular level is crucial for stepping forward in electromethanogenesis through genetic engineering of methanogenic archaea [15]. In the present work, we aimed at studying changes in the expression of genes coding for selected subunits of membrane-bound Eha and Ehb hydrogenases, cytoplasmic Hdr complex and hydrogenase maturation protein HypD, in naturally enriched cultures of methanogenic archaea. Experiments were conducted in electromethanogenic reactors built *ad hoc* for the present experiments. *Methanobacterium* sp. was the main responsible archaeon for electromethanogenesis in our system. Changes from closed and open electric circuits were used to analyze the relative expression of the selected genes.

## Material and methods

### Configuration and operation of the bioelectrochemical system

A bioelectrochemical system (BES) was set up in house using a three-neck round bottom flask (Duran-Group, Germany) with a nominal capacity of 1 L. The anodic and cathodic chambers were set at working volumes of 15 and 940 mL, respectively. Anodic and cathodic chambers were separated by a cationic exchange membrane (CMI-7000, Membranes International Inc, USA). The BES contained four cathodes made of carbon cloth (NuVant’s ELAT LT2400W, FuelCellsEtc, USA) each one with a 42 cm^2^ surface area. Cathodes were independently connected to a potentiostat through a stainless steel wire (S1 Fig). Biocathodes were used as working electrodes operated chronoamperometrically at– 800 mV *vs*. SHE to guarantee hydrogen production [3]. A carbon rod (5x250 mm, MERSEN IBERICA, Spain) was used as a sacrificial anode. The Ag/AgCl reference electrode (+197 mV *vs*. SHE, sat KCl, SE11 Sensortechnik Meinsberg, Germany) was placed in the cathode chamber. Precision rubber septa tightly closed the reactor compartments. Current demand was monitored by means of a potentiostat (BioLogic, Model VSP, France).

Cathode and anode chambers were filled with mineral medium which contained (concentration per liter): 1 g KH_2_PO_4_, 1 g NaCl, 0.25 g NH_4_Cl, 0.05 g MgCl_2_, 0.1 g KCl, 0.03 g CaCl_2_. The medium was supplemented with 1 mL·L^-1^ of trace metal solution (concentration per liter: 20 g nitrilotriacetic acid, 10 g MnSO_4_·H_2_O, 8 g Fe(SO_4_)_2_(NH_4_)_2_·6H_2_O, 2 g CoCl_2_·6H_2_O, 0.002 g ZnSO_4_·7H_2_O, 0.2 g CuCl_2_·2H_2_O, 0.2 g NiCl_2_·2H_2_O, 0.2 g Na_2_MoO_4_·2H_2_O, 0.2 g Na_2_SeO_4_, 0.2 g Na_2_WO_4_) and 1 mL·L^-1^ of vitamin solution (concentration per liter: 20 mg biotin, 20 mg folic acid, 100 mg pyridoxine hydrochloride, 50 mg thiamine hydrochloride, 50 mg riboflavin, 50 mg nicotinic acid, 50 mg DL- calcium pantothenate, 1 mg vitamin B12, 50 mg p- aminobenzoic acid, 50 mg lipoic acid). Prior to the inoculation of the system, cathodic and anodic chambers were bubbled with pure CO_2_ (<99.95%, Praxair, Spain) for at least 10 minutes.

The BES was inoculated with the effluent of a parent electromethanogenic reactor operated according to Batlle-Vilanova et al.[3]. The parent reactor exhibited a constant methane production at the time of effluent sample collection. Two samples were collected at different position in the reactor and mixed at equal volumes to inoculate the BES used here. Inoculation was done at a 1/10 ratio. The BES was kept at constant stirring at 37±1°C, atmospheric pressure and was wrapped in aluminum foil to restrict phototrophic activity. The BES was operated chronoamperometrically (closed electric circuit) for 53 days until methane production rates and current demand were maintained constant for at least one week. Once these conditions were achieved, two of the electrodes were disconnected (open electric circuit; named as Cat1 and Cat2) and maintained for up to six hours to test for changes in gene expression. The other two electrodes remained connected to the electric circuit (closed electric circuit). Six hours incubation was chosen in order to detect sudden changes in gene expression levels in the event of a restriction on the electron availability, and to minimize potential side effects due to H_2_ production (biotically or abiotically) in the biofilm. Unfortunately, experiments could not be repeated for longer incubation times due to the sacrificial sampling strategy of the cathode. After this period of time, the four electrodes were removed, cut into small pieces using RNAse free forceps and scissors, and the samples were collected subsequently for molecular analyses. Samples for RNA extraction (approximately, 30 cm^2^) were immediately frozen in liquid nitrogen. Samples for DNA extraction (approximately, 8 cm^2^) were maintained at -20°C. The rest of the cathode material was preserved to analyze the biofilm structure by scanning electron microscopy.

### Electrochemical analyses

Electrochemical performance of each cathode was assayed independently using cyclic voltammetry (CV) with the software EC-Lab v10.37 (Bio-Logic Science Instruments, France). CVs were performed under turnover conditions shortly before the end of the incubation period (days 34 to 38) to characterize the microbial electrochemical activity. CV signals for each electrode were compared to recordings in abiotic conditions collected after the set-up of the reactor and before the inoculation (day 1). Data extracted from CVs as peak detection and first derivative analyses were performed using the free-software SOAS [16]. The mid-point potential (Ef) of redox couples was calculated as the mean value of the oxidative and reductive potential.

Gas and liquid samples were taken periodically (on average, twice per week) from the cathode compartment to monitor pH, conductivity, and the composition of gas and liquid phases. Gas composition was analyzed using an Agilent 7890A (Agilent Technologies, USA) gas chromatographer (GC) to analyze CH_4_, CO_2_, carbon monoxide (CO), oxygen (O_2_) and H_2_. Volatile fatty acids (i.e. acetate) and alcohols (i.e. ethanol) were also analyzed in the same GC. Conductivity and pH were measured with an EC Meter Basic 20^+^ and a pH-Meter Basic 20^+^ (Crison Instruments, Spain), respectively. In order to keep a constant volume in the reactor, withdrawn volumes during sampling were replaced with freshly prepared medium. After sampling, pure CO_2_ (>99.95%, Praxair, Spain) was bubbled for over five minutes to ensure CO_2_ saturation in the medium.

### Extraction of DNA and RNA

DNA was extracted from bulk liquid and biofilm samples. For the bulk liquid samples, cells were pelleted by centrifugation prior to DNA extraction, whereas carbon cloth samples were used directly for biofilm DNA extraction. DNA was extracted using the FastDNA SPIN kit for Soils (MP Biomedicals, USA) following the manufacturer’s instructions. The extracts were distributed in aliquots and stored at -20°C. DNA concentration was measured using a Nanodrop 1,000 spectrophotometer (Thermo Fisher Scientific, USA).

RNA was extracted from biocathodes using TRIzol Max Bacterial RNA Isolation Kit (Ambion, USA). Lysis of cells was performed in a bead-beater. Carbon cloth samples were placed in bead tubes (0.3 g zirconia beads, 0.1 mm diameter) followed by the addition of 200 μL of MAX Bacterial Enhancer (Ambion, USA). Tubes were incubated at 95°C for 4 minutes. After this incubation Trizol (1 mL) was added and samples were incubated for 5 minutes at room temperature, before being shaken at maximum speed using a Mo-Bio Vortex Genie 2 (Mo-Bio Laboratories, Inc., USA) for 15 minutes. RNA extracts were quantified using the Agilent 2100 Bioanalyzer (Agilent Technologies, USA). Co-extracted DNA was cleaned up from RNA extracts by using the DNase digestion and RNA Cleanup protocols of RNeasy Mini Kit (QIAGEN, Germany). cDNA was synthesized with the High Capacity cDNA Reverse Transcription kit (Applied Biosystems, USA) according to the manufacturer’s instructions. 10 μL of digested RNA extracts at a minimum concentration of 5 ng/μL were used in all cases.

Quality of DNA and cDNA extracts for downstream molecular applications were checked after PCR detection of 16S rRNA using universal bacterial primers 357F and 907R.

### Microbial community structure determination

The hypervariable V4 region of the 16S rRNA gene for both DNA and cDNA samples was amplified using the primers 515F - 806R and method described by Kozich and Schloss, adapted to produce a dual-indexed Illumina compatible libraries in a single PCR step [17]. Primary PCR was performed using fusion primers with target-specific portions [18], and Fluidigm CS oligos at their 5' ends. Secondary, PCR targeting the CS oligos was used to add sequences necessary for Illumina sequencing and unique indexes. PCR products were normalized using Invitrogen SequalPrep DNA normalization plates. The pooled samples were sequenced using an Illumina MiSeq flow cell (v2) using a 500-cycle reagent kit (2x250bp paired-end reads). Sequencing was done at the RTSF Core facilities at the Michigan State University USA (https://rtsf.natsci.msu.edu/).

Paired-end sequences were merged, quality filtered and clustered into OTUs (Operational Taxonomic Units) using USEARCH v9.1.13 [19]. Sequences were filtered for minimum length (>250 nt) and maximum expected errors (<0.25). OTUs were clustered at the 97% identity using UCLUST [20], and checked for the presence of chimeras. OTUs containing only one sequence (singletons) were removed. The subsequent analyses were performed with Qiime v1.9.1 [21]. Representative OTUs sequences were aligned using PyNAST with default parameters against Silva 128 release (February 2017). The same reference database was used to taxonomically classify the representative sequences using UCLUST. Direct BLASTn searches at the NCBI of selected sequences were used when poor identifications with the Silva database were obtained.

Richness indicators, i.e. the number of different species in the sample, were calculated as the observed (Sobs) and maximum estimates thus showing the number of species at 100% coverage (Chao1). Diversity indicators, i.e. a measure of the relative abundance and composition of microbial species in the sample, were calculated as Shannon and Phylo-diversity indices. These indicators were calculated only for DNA samples using randomly collected subsets of 61,000 sequences per sample. Ten iterations were performed and mean values calculated.

Beta diversity was only calculated for biofilm samples (DNA and cDNA based analyses) using randomly collected subsets of 21,000 sequences. Unweighted and weighted UniFrac distances were calculated to compare the microbial community structure between samples [22]. Weighted UniFrac distances were used for the jackknife-resampling analysis. Differences in the community structure were visualized either as a dendrogram or a Principal Coordinates plot. Dendrogram of sample distributions generated in QIIME was visualized in the Interactive Tree of Life software [23]. Groups of samples were statistically compared using ANOSIM.

Significant differences in OTU abundance between groups (DNA *vs*. cDNA based communities or open *vs*. closed electric circuits) was analyzed using non-parametric t-tests with QIIME, and based on rarefied OTU tables for each sample.

The sequences presented in this study have been submitted to the GenBank database within the SRA accession number SRP153784 (BioProject ID PRJNA481232).

### RT-PCR evaluation of hydrogenase genes

Sequences, for selected genes coding for subunits of the hydrogenase complexes or maturation proteins (*ehaB*, *ehbL*, *mvhA*, *hdrA*, *frhA*, and *hypD*), formyl-methanofuran dehydrogenase (*fwdD*), formate dehydrogenase (*fdhB*), housekeeping (*ftsZ*) and 16S rRNA genes of members of the genus *Methanobacterium* were retrieved from the NCBI database. Representative sequences for each gene were aligned using Bioedit (Biological sequence alignment editor v7.2.6), and conserved regions were primarily searched for suitable specific degenerated PCR primers. Primers were designed using the Primer-BLAST design tool [24]. The following parameters were used: amplicon size was limited to 300 bp, and predicted melting temperatures were set between 57°C and 65°C. *In silico* predictions of primer specificity were performed against the nr database of the NCBI, and organism choice were restricted to *Methanobacterium*. Primers with the least self-complementarity value and minimum difference between melting temperatures were selected (S1 Table). Primer specificity towards *Methanobacterium* was checked using DNA extracts of other microorganisms, *Archaeoglobus fulgidus* DSM 4304, *Halobacterium salinarum* DSM 3754, *Thermoplasma acidophilum* DSM 1728, *Methanobacterium alcaliphilum* DSM 3387, *Sulfolobus solfataricus* DSM 1616, *Methylobacterium extorquens* DSM 1337 and *Methylomonas methanica* NCIMB 1130. Changes of annealing temperature and MgCl_2_ concentrations were applied when necessary for PCR optimization. No product specificity towards *fwdD* and *fdhB* resulted with the designed primers for *Methanobacterium* sp. PCR reactions were run at GeneAmp PCR System 2,700 (Applied Biosystems, USA) with Ampli Taq 360 (Applied Biosystems, USA). Optimized reaction conditions were used for semi-quantitative RT-PCR using the LightCycler 480 SYBR Green I Master (Roche Life Science, Switzerland). RT-PCR reactions were run in a Lightcycler 96 Real-Time PCR system. Two sample volumes, 1 and 2 μL in a 20 μL total volume were used to ensure no inhibition occurred. In all cases, conventional and quantitative RT-PCR, amplicons were visualized in agarose (2%) gel. Additionally, melting curves were also recorded for quantitative RT-PCR amplifications.

Standard curves for each set of primers were calculated using sequential dilutions from a DNA sample (1:10 to 1:50000). Quantitative efficiencies were slightly high, specifically at the low concentration range (S1 Table). Numbers of copies per each gene were estimated from calibration curves. To facilitate comparison between open and closed conditions, relative gene concentrations were calculated (copies of gene/copies of 16S rRNA). Changes in the relative concentration of each hydrogenase gene were tested for statistical significance using a non-parametric (U Mann-Whitney test).

## Results and discussion

### Bioelectrochemical performance and methane production

**Fig 1:**
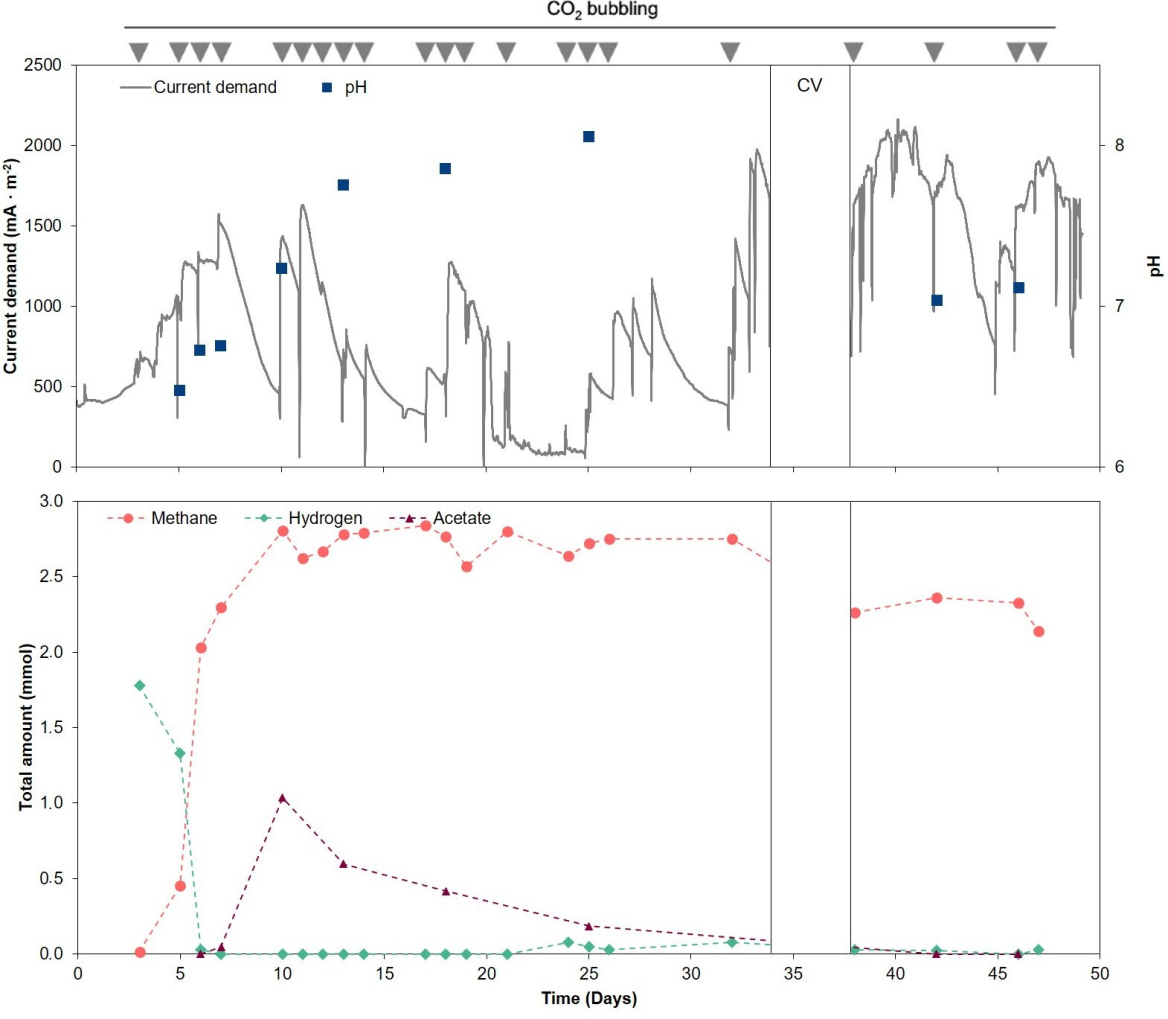
Time course of state variables during the operation time of the BES reactor (53 days). Upper plot- Current density and pH variation. Lower plot- Methane, hydrogen and acetate amounts (mmol). Methane and hydrogen were calculated as added amounts considering the liquid and gas phases. CO_2_ flushes are indicated with grey triangles on top of the graph.

The electromethanogenic system was operated for 53 days feeding CO_2_ as the sole carbon source. Hydrogen was only recorded at the beginning of the experiment, 1.78 mmols (73.5% v/v in the headspace) and 1.33 mmols (54.8% v/v) at days 3 and 5, respectively, and remained below detection limits (< 2% v/v) for most of the time (Fig 1). Low hydrogen concentration indicated a high consumption rate for methanogenesis. Volumetric concentration of methane remained from 70 to 90% (v/v), reaching its maximum at day 17 where the concentration was 2.77 mmols (98.8% v/v in the headspace). Invariably, at the time gas samples were collected CO_2_ was detected at a 10 to 30% (v/v) in the headspace, indicating that CO_2_ was not limiting for methanogenesis in the used conditions. Oxygen was detected occasionally (<5%) in the cathode and was most likely due to diffusion from the anode compartment. To avoid this accumulation, extended CO_2_ bubbling times were used. As previously shown, oxygen is an effective methanogenesis inhibitor at rather low concentrations [25]. Acetate presence was minimal (maximum concentration of 1.0 mM on day 10).

Cyclic voltammetries (CV) were performed before inoculation of the system and on day 34 for the four biocathodes independently (S2 Fig). A redox pair was clearly identified at -0.49 ± 0.06 V *vs*. SHE at 37°C, which is within the range of redox potential values generally assigned to the activity of hydrogenases [26]. This redox pair was also observed in H_2_-producing biocathodes, in which an active biofilm was suspected to catalyze a biotic hydrogen evolution, decreasing the important energy losses associated to catalytic hydrogen production [3,27]. This decrease in the redox pair implies that reduction is kinetically favored and accordingly, hydrogen could be produced more energy efficiently. In other studies, is shown that microorganisms can act as catalysts producing hydrogen but consuming almost half of the energy when compared with an abiotic cathode [3].

### DNA based microbial community structure

The presence of a dense biofilm attached to the electrode surface was clearly visualized by SEM observations (S1 File). The majority of microbes attached to the carbon cloth were rod-shaped and thin appendage-like structures, between cells and cathode surface were sporadically observed (S3 Fig).

The microbial community structure was analyzed by sequencing a 250 bp fragment of the 16S rRNA. A total of 854,427 valid sequences were obtained. The average number of sequences per sample was 71,202 (from 63,505 to 144,017). One of the bulk liquid samples was removed due to a limited sequencing depth. Sequences were clustered in 443 OTUs at a similarity level of 97%. Each sample was rarefied at 61,000 sequences to analyze changes in the composition of the microbial community. For all samples coverage was higher than 99%. Richness and diversity indicators were invariably lower in the BES compared to the sample used as inoculum (Table 1), showing a selective enrichment of species in the cathode biofilms (lower number of species).

**Table 1:** Richness and diversity indicators according to sample type.

	Inoculum	Biofilm	Bulk liquid
**Observed Richness (Sobs)**	201.2 ± 9.6	117.5 ± 27.1	158.7
**Maximum richness (Chao1)**	205.6 ± 10.9	134.9 ± 28.7	172.7
**Shannon diversity (H’)**	4.5 ± 0.5	2.8 ± 0.7	3.9
**Phylodiversity (PD)**	11.4 ± 0.4	7.9 ± 1.1	9.9

**Fig 2:**
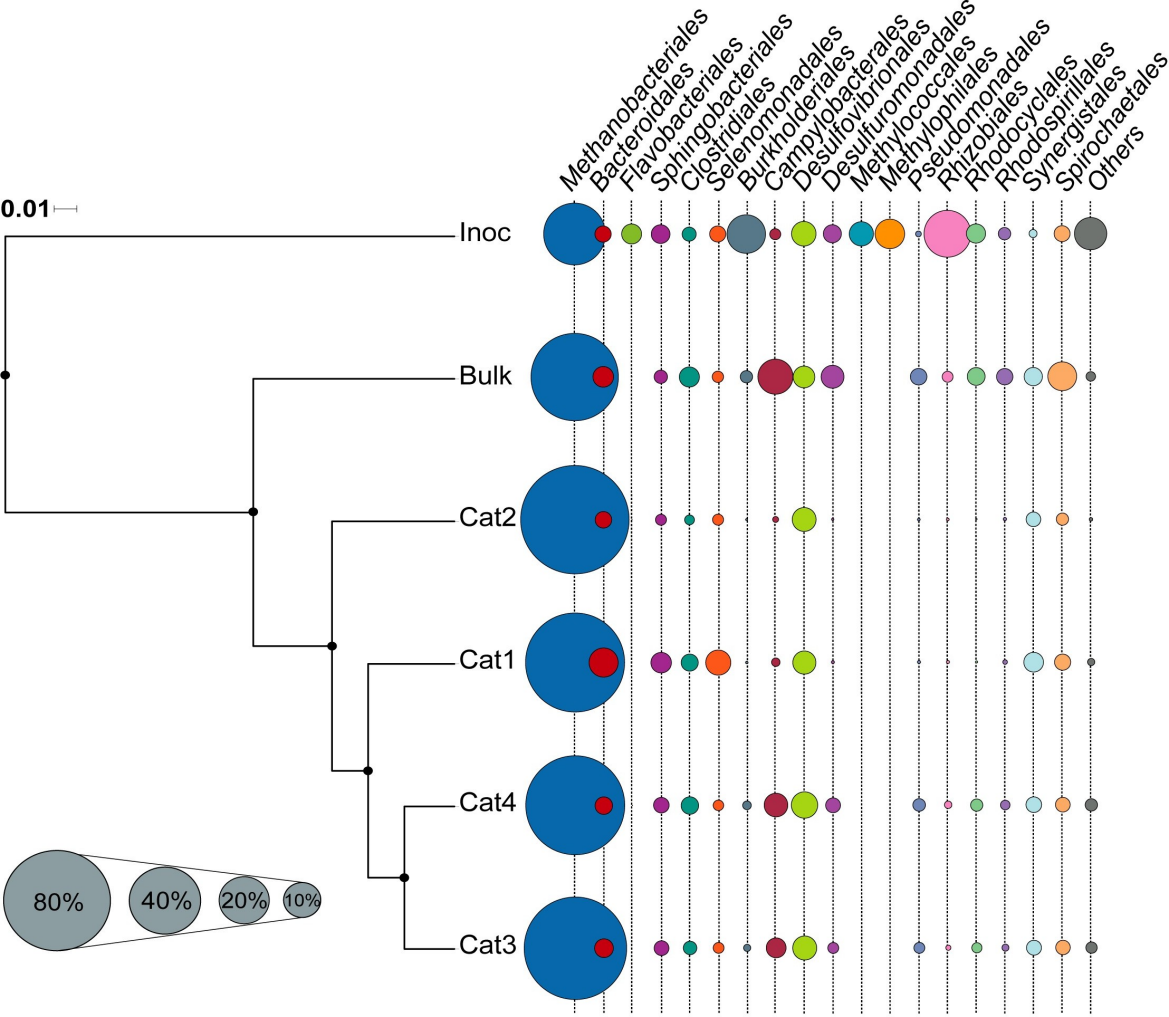
Microbial community composition in inoculum, bulk and biofilm samples. Dendrogram based on weighted Unifrac measures of the microbial community rarefied at 61,000 sequences per sample. Black dots in nodes indicated bootstrap supported levels above 90%. Bubble chart show the relative abundance of main archaeal and bacterial orders in each sample. Phyla accounting for less than 0.01% of all the sequences have been grouped as Others. Cat1, Cat2, Cat3 and Cat4, are samples from the biofilm of the four carbon cloth cathodes.

BES biofilms resulted in a highly enriched population of methanogenic archaea (75.7% of sequences in the biofilm) compared to bulk liquid (56.7%) (Fig 2). *Proteobacteria* showed an opposite trend and were more abundant in the bulk liquid (8.9% and 25.7% of the sequences in biofilm and bulk liquid samples, respectively). Despite the higher enrichment of methanogenic organisms in the biofilm, no significant differences in overall community structure were detected (R = 0.92, p > 0.05).

At the genus level, BES was clearly dominated by *Methanobacterium* spp. BLASTn searches of the most abundant OTUs resulted in the presence of sequences highly similar to *M*. *congolense* and *M*. *formicicum* (Table 2). Other methanogens, such as *Methanobrevibacter* sp., were represented at lower relative abundances (<2% considering all sequences).

**Table 2:** Most probable identification (BLAST, refseq rna database), and relative abundance of the two most abundant archaeal 16S rRNA sequences in the electromethanogenic reactor.

Most probable identification	Similarity (%)	Relative number of sequences (%)		
		Inoculum	Biofilm	Bulk liquid
*Methanobacterium congolense* strain C NR_028175.1	99	2.9 ± 2.2	49.6 ± 19.3	31.4
*Methanobacterium formicicum* strain MF NR_115168.1	100	25.6 ± 18.3	12.6 ± 7.8	15

Bacterial members of the microbial community distributed in different abundances between biofilm and bulk liquid communities (Fig 2). Interestingly, members with an active sulfur metabolism, i.e. *Desulfovibrio* and *Sulfurospirillum*, were detected in all samples analyzed. *Desulfovibrio* sp. was present at relatively higher densities in all four cathodes. They could be contributing to the bioelectrically mediated production of hydrogen, as an intermediate step to methanogenesis, like previously reported [2,28]. Biotic H_2_ production has been demonstrated in biocathodes when these sulfate-reducing bacteria are present [29,30].

### Determination of active members of the microbial community by RNA analysis

Dominance of *Methanobacterium* in BES has been previously reported in studies conducting electromethanogenesis where both hydrogen-mediated and direct electromethanogenesis were supposed to occur [1–3,28]. All *Methanobacterium* spp. have been considered as hydrogenotrophic methanogens [31], and more specifically *Methanobacterium palustre* has been suggested as being responsible for direct electron transfer in a cathode [1]. However, the applied potentials in Cheng’s et al. work (from -0.5 to -1.0 V *vs*. SHE) did not guarantee that hydrogen mediated methanogenesis was not taking place simultaneously. Abiotic hydrogen production in graphite electrodes has been studied at cathodic voltages ranging -0.4 to -1.8 V *vs*. SHE and it was only detected at potentials below -0.9 V *vs*. SHE [32]. Similar voltage ranges (below -0.8 V *vs*. SHE) were recorded as the threshold for significant abiotic hydrogen evolution for carbon cloth cathodes [33]. These values have been confirmed experimentally with the same systems used here (unpublished results). Although abiotic hydrogen evolution cannot be overridden in the present system, it should have remained at low rates. Moreover, H_2_ was not detected above detection limit in any of the gas samples analyzed, suggesting a high consumption rate, and limiting H_2_ availability at the used conditions.

**Fig 3:**
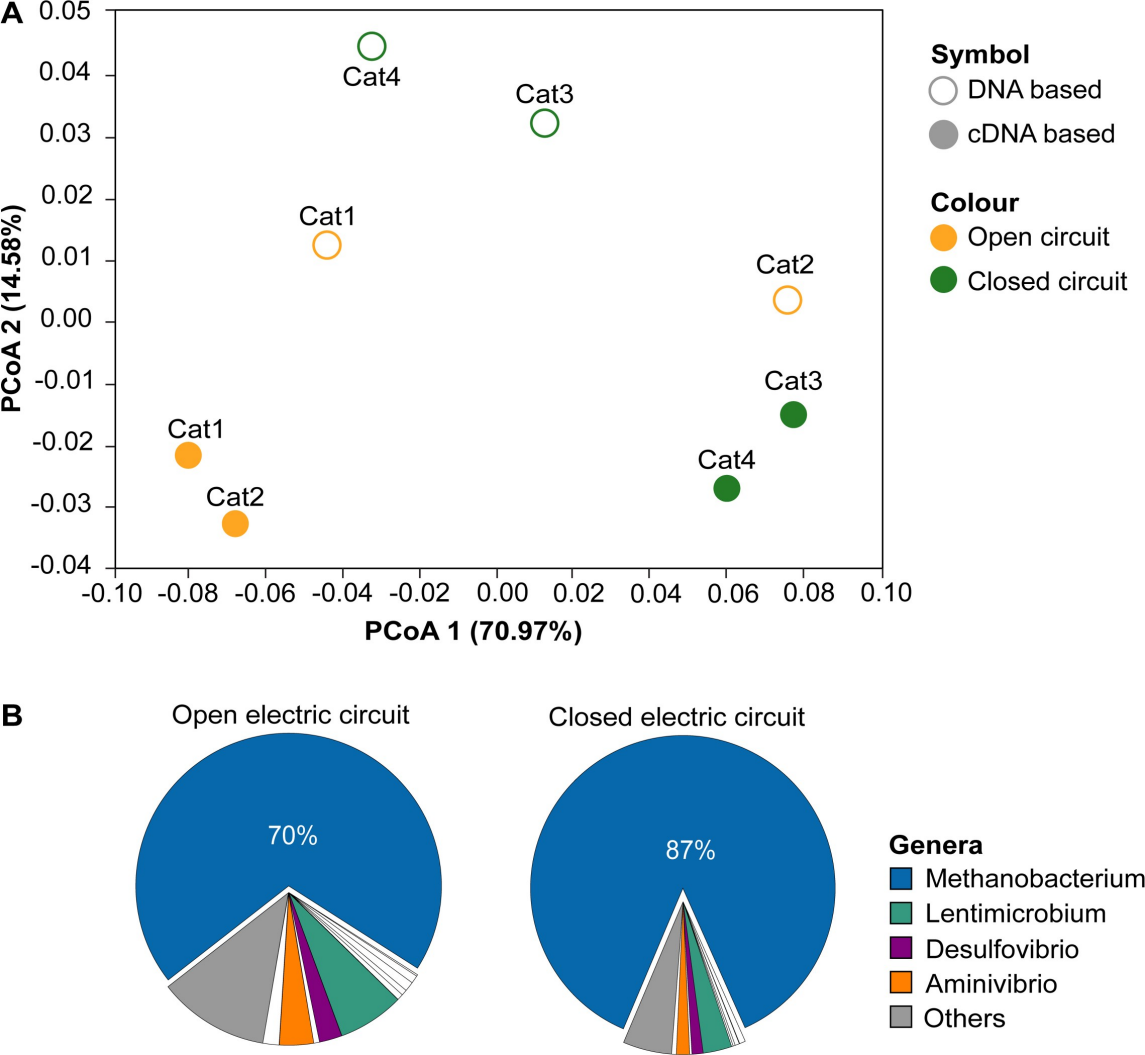
Changes in the DNA and cDNA based microbial community structures of cathodes. (A) Distribution of samples in a Principal coordinates analysis plot (PCoA) according to a weighted Unifrac dissimilarity index using 21,000 sequences per sample (see text for details). The variance (%) explained by each axis is indicated. (B) Pie charts of the average microbial composition (relative number of sequences) for the cDNA based community of open and closed circuits.

cDNA analysis was used as a proxy to identify the active members in the biofilm community, in both open and closed electric circuit cathodes. *Methanobacterium* sp. related sequences (70.0% to 87.0% of sequences) were dominant in the active community (Fig 3).

As described before, the sensitivity of electromethanogenic biofilms to the electrode-to-cell flow showed a negative effect in methane production rates when reactors were exposed to an open electric circuit (cathode was disconnected from the potentiostat), suggesting that current was the unique energy source for methanogenesis [3].

Clearly, *Methanobacterium* was the main responsible archaeon conducting electromethanogenesis in the studied set-up. This archaeon, being the most abundant, and in an active state, was used as a model to study if cathode poising was a key factor in the expression of *Methanobacterium* membrane-bound and soluble [NiFe]-hydrogenases involved in the electron transfer chain during methanogenesis.

### Changes in the expression of *Methanobacterium* sp. [NiFe]-hydrogenases

The role of [NiFe]-hydrogenases was tested as proteins putatively implicated in electron transfer mechanism in *Methanobacterium* sp. We designed specific primers for key components of the enzyme complexes and tested for changes in their relative abundance using quantitative RT-PCR.

**Fig 4:**
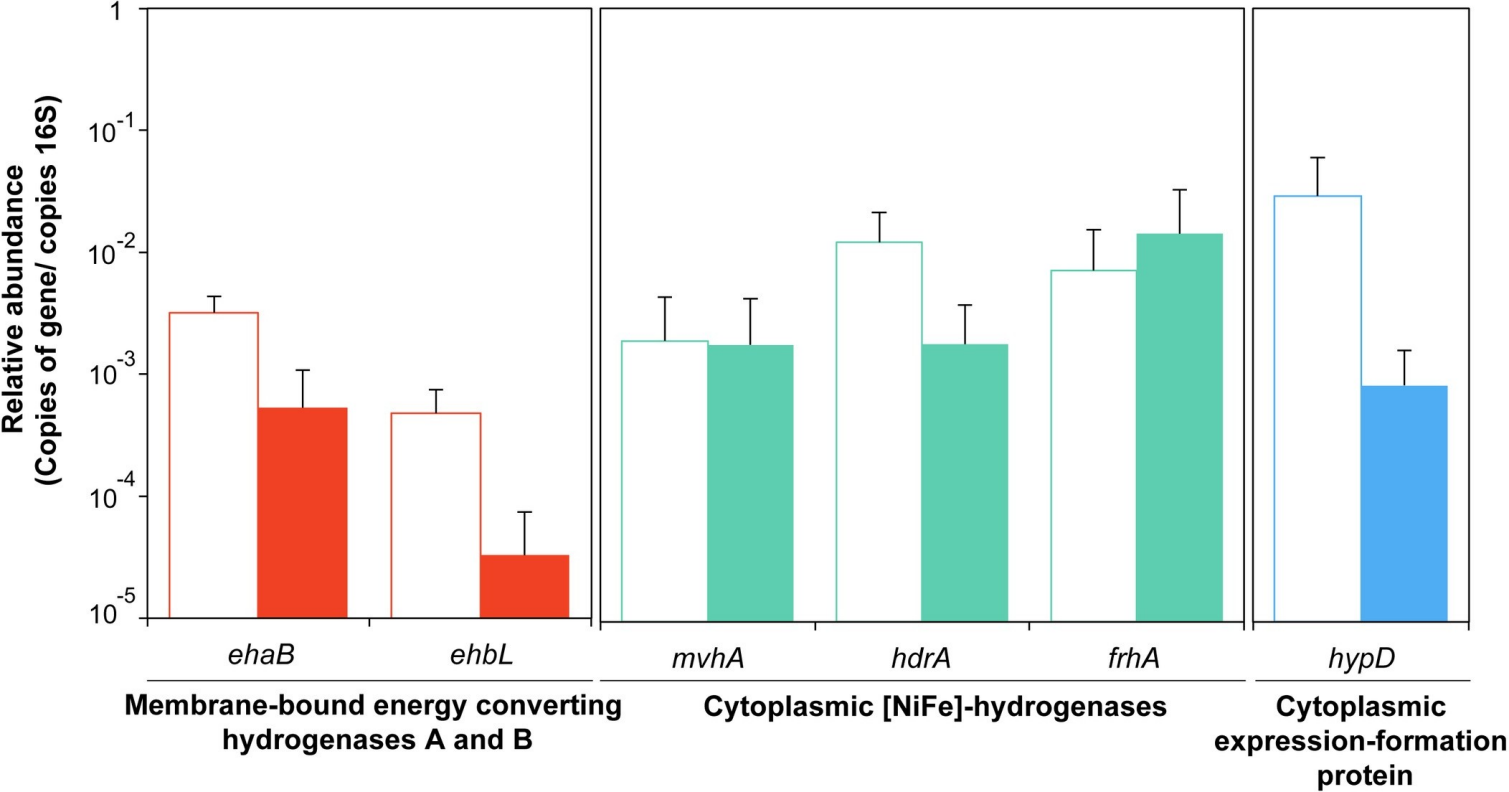
Relative content (number of target gene copies/number of copies 16S rRNA gene) of *Methanobacterium* sp. hydrogenases related genes. Open (empty bars) and closed (filled bars) electric circuit conditions were analyzed. Mean values and standard deviations are represented in the bar chart. Relative abundance is expressed using logarithmic scale. *ehaB*–energy-converting hydrogenase A subunit B. *ehbL*–energy-converting hydrogenase B subunit L. *mvhA*—heterodisulfide reductase associated [NiFe]-hydrogenase subunit A. *hdrA*—heterodisulfide reductase subunit A. *frhA*—Coenzyme F_420_-reducing [NiFe]-hydrogenase subunit A. *hypD*–hydrogenase formation protein hypD.

Primers were directed to subunits containing [NiFe] or [Fe-S] clusters. Unfortunately, no reliable primer pairs could be obtained for the active subunit of Eha and we considered using primers for subunit *ehaB* being representative of hydrogenase expression since they are transcribed as a single operon [12]. Albeit not significant (non-parametric U Mann-Whitney test, p-value > 0.05), the observed relative expression of hydrogenases pointed to an increase of the relative expression of four of the genes, *ehaB*, *ehbL*, *hdrA*, and *hypD*, accounting for increases up to 1.5-fold when electrodes were disconnected (Fig 4). Similar results were obtained when relative expression was calculated according to *ftsZ* as the housekeeping gene for normalization (S4 Fig).

We hypothesized a change in the relative expression levels of genes coding for enzymes participating in direct electron capturing would occur after disconnection of the electric circuit, if the process was regulated at the transcriptomic level. With the used BES configuration, hydrogen mediated and direct electromethanogenesis was supposed to occur concomitantly, therefore at open circuit conditions, availability of reducing equivalents would have been reduced immediately, and a change on the expression of selected hydrogenases would be expected in the event of their participation in direct electron capturing. The observed increase in the relative number of transcripts of the selected genes at disconnected conditions pointed to a more severe hydrogen limitation.

Bioavailability of H_2_ affects several physiological and regulatory processes, altering the expression of some of the key genes for methanogenesis [13]. For instance, H_2_ limiting conditions in *M*. *maripaludis*, resulted in a higher expression of coenzyme F_420_ [NiFe]-hydrogenase (Frh), a moderately up-regulation of Ehb, whereas no significantly effect was detected in the expression of Eha and heterodisulfide reductases (Hdr) [34]. In the event of electrons causing a similar effect as H_2_ limitation in cells, we expected an up-regulation of Ehb and Frh. In contrast, we did not observe any significantly expression differences analyzing *ehbL* and *frhA* in *Methanobacterium* sp. *ehaB*, *mvhA*, *hdrA* and *hypD* also remained unchanged.

The reduced changes of the relative expression of *Methanobacterium* sp. hydrogenases when shifted from connected to disconnected circuits can be due to a combination of different aspects. First, the hydrogen produced at the electrodes that remained connected to the current (located in the same reactor) would maintain minimum levels of available reducing equivalents preventing significant changes in the gene expression. Second, six-hour exposure to the open electric circuit was not sufficient to trigger significant changes in expression levels of the target genes, which would have been facilitated in the presence of a H_2_ sensory apparatus. Regulating H_2_-sensors are absent in *M*. *marburgensis* and *M*. *thermoautotrophicus* genomes [35], suggesting that longer exposure times to open electric circuits should be tested to confirm changes in the relative, expression of hydrogenase genes. Third, the tested enzymes were not really participating in electron capture in *Methanobacterium* sp., or they were not sensitive to changes in the electron availability.

In a previous report, Lohner and co-workers suggested the presence of a hydrogenase-independent mechanism of electron catabolism with the archaeon *Methanococcus maripaludis* MM1284. MM1284 is a mutant strain, carrying deletions of six hydrogenase genes, and is unable to perform methanogenesis from CO_2_ and H_2_. Bioelectrochemically mediated methane production of MM1284 was observed at cathode potentials of -600 mV and -700 mV *vs*. SHE, suggesting the existence of a hydrogenase-independent electron uptake mechanism later shown to be involving formate by Deutzmann and co-workers [4,36]. According to our results, participation of *Methanobacterium* sp. hydrogenases (Ehb and Eha) in electron capture could not be confirmed.

More recently, the participation of heterodisulfide reductase complex has also been reported as a facilitator for electromethanogenesis in *M*. *maripaludis* [6]. We analyzed three *Methanobacterium* sp. proteins forming heterodisulfide complexes. Transcripts of *hdrA*, *mvhA* and *frhA* seemed to be unaffected when cells were shifted from connected to disconnected conditions. Unfortunately, formate dehydrogenase (Fdh) and formyl-methanofuran dehydrogenase (Fwd) could not be analyzed due to the lack of specificity of the primers for *Methanobacterium* sp..

The majority of genes coding for proteins highlighted as putative electron harvesting proteins have been investigated in this work, and changes in their relative expression analyzed in a highly enriched *Methanobacterium* population. Our results point to a slight increase on the relative expression of four of these genes (*ehaB*, *ehbL hdrA* and *hypD*) although differences were not conclusive with the conditions used here. Additional tests need to be performed in order to confirm the observed tendency and to incorporate other proteins, such as ferredoxins [37], or pili proteins [38] that are likely to be involved in electrode capturing and deserve further investigation in electromethanogenesis.

## Supporting information

S1 Table Designed primers. Target gene, primer name, sequence and annealing temperature are shown. Underlined degenerated positions. (ND. Not determined).
(DOCX)

S1 Fig Bioelectrochemical system (BES) configuration. (TIF)

S2 Fig Electrochemical performance of each cathode was assayed independently using CV after 34 days of operation. Cyclic voltammetry (CV) tests for each electrode (above) and first derivative of the respective CVs (below) under abiotic (grey) and biotic (black) conditions.
(TIF)

S1 File Supplementary Methods. (DOCX)

S3 Fig Scanning electron micrograph of the biofilm attached to the cathodes. (A) Colonized carbon cloth fiber. (B) Detail of rod shaped microbes. (C) Thin appendage-like structures (white arrows) between microorganisms and carbon cloth surface were observed.
(TIF)

S4 Fig Relative content (number of target gene copies/number of copies of *ftsZ* gene) of *Methanobacterium* sp. hydrogenases related genes. Open (empty bars) and closed (filled bars) electric circuit conditions were analyzed. Mean values and standard deviations are represented in the bar chart. Relative abundance is expressed using logarithmic scale. *ehaB*–energy-converting hydrogenase A subunit B. *ehbL*–energy-converting hydrogenase B subunit L. *mvhA*—heterodisulfide reductase associated [NiFe]-hydrogenase subunit A. *hdrA*—heterodisulfide reductase subunit A. *frhA*—Coenzyme F_420_-reducing [NiFe]-hydrogenase subunit A. *hypD*–hydrogenase formation protein hypD.
(TIF)
