# Supplementary material for: [NiFe]-hydrogenases are constitutively expressed in an enriched Methanobacterium sp. population during electromethanogenesis
Source: PLoS One. 2019 Apr 11;14(4):e0215029. doi: 10.1371/journal.pone.0215029 (PMC6459506; doi:10.1371/journal.pone.0215029)
Supplement: S1 Table — Target gene, primer name, sequence and annealing temperature are shown. Underlined degenerated positions. (ND. Not determined). (DOCX) [file pone.0215029.s001.docx]

| **Gene** | **Primer** | **Sequence (5’-3’)** | **Annealing temperature (ºC)** | **Expected amplicon size (bp)** | **Primer efficiencies (%)** | **Reference** |
| --- | --- | --- | --- | --- | --- | --- |
| Heterodisulfide reductase associated [NiFe]-hydrogenase subunit A *mvhA* | mvhA-F | AGGAAAYGTYSAGGACACCAA | 57 | 263 | 204 | This study |
|  | mvhA-R | AGTAGAAGTGVAGWGCGTGG |  |  |  |  |
| Heterodisulfide reductase subunit A *hdrA* | hdrA-F | CATCCCATTCCCACARGCA | 57 | 183 | 165 | This study |
|  | hdrA-R | GTTGGGTYGTATGGGTCGT |  |  |  |  |
| Coenzyme F_420_-reducing [NiFe]-hydrogenase subunit A *frhA* | frhA-F | TAGTATHAGTCCTGTTAGAGGTT | 57 | 204 | 190 | This study |
|  | frhA-R | ATGGTRTGGGCAGCAAGAGT |  |  |  |  |
| Energy-converting hydrogenase A subunit B *ehaB* | ehaB-F | CTTTGGTAAYMGGCTGTGCG | 60 | 151 | 167 | This study |
|  | ehaB-R | GCCCTGGATGGTAAGGATGG |  |  |  |  |
| Energy-converting hydrogenase B subunit L *ehbL* | ehbL-F | ATTGGCTGCGGRGGWTGCAG | 62 | 200 | 126 | This study |
|  | ehbL-R | GGGTGTATGGTTCCTGCCTG |  |  |  |  |
| Hydrogenase expression-formation protein HypD | hypD-F | GTCTGCGGATCMCATGAACA | 60 | 109 | 147 | This study |
|  | hypD-R | CGGGTACACAGCABACHGGA |  |  |  |  |
| Formyl-mehtnaofuran dehydrogenase subunit D *fwdD* | fwdD-F | AAAGGTAKGTGGAGTTGCTT | ND | 207 | ND | This study |
|  | fwdD-R | CAGCAGCAGTAGGTTTCGTG |  |  |  |  |
| Formate dehydrogenase subunit B *fdhB* | fdhB-F | CTTYGTCTTCCCAATC*K*TTACCTT | ND | 160 | ND | This study |
|  | fdhB-R | TGTTCCCCTAAAGGTGYTGAA |  |  |  |  |
| rRNA subunit 16S | Arc915f | AGGAATTGGCGGGGGAGCAC | 63 | 283 | 156 | [1] |
|  | MB1174 | TACCGTCGTCCACTCCTTCCTC |  |  |  | [2] |
| Cell division protein FtsZ | ftsZ-F | CTGGGAAGGGCWGTDAAGGG | 60 | 122 | 172 | This study |
|  | ftsZ-R | TCACCATTCCKATCATKGC |  |  |  |  |

**References**

1. Raskin L, Stromley JM, Rittmann BE, Stahl D a. Group-Specific 16S Ribosomal-Rna Hybridization Probes To Describe Natural Communities of Methanogens. Appl Environ Microbiol. 1994;60: 1232–1240. doi:0099-2240/94/$04.00+0

2. Cheng S, Xing D, Call DF, Logan BE. Direct Biological Conversion of Electrical Current into Methane by Electromethanogenesis. Environ Sci Technol. 2009;43: 3953–3958. doi:10.1021/es803531g
