# Supplementary figures and images for: [NiFe]-hydrogenases are constitutively expressed in an enriched Methanobacterium sp. population during electromethanogenesis
Source: PLoS One. 2019 Apr 11;14(4):e0215029. doi: 10.1371/journal.pone.0215029 (PMC6459506; doi:10.1371/journal.pone.0215029)

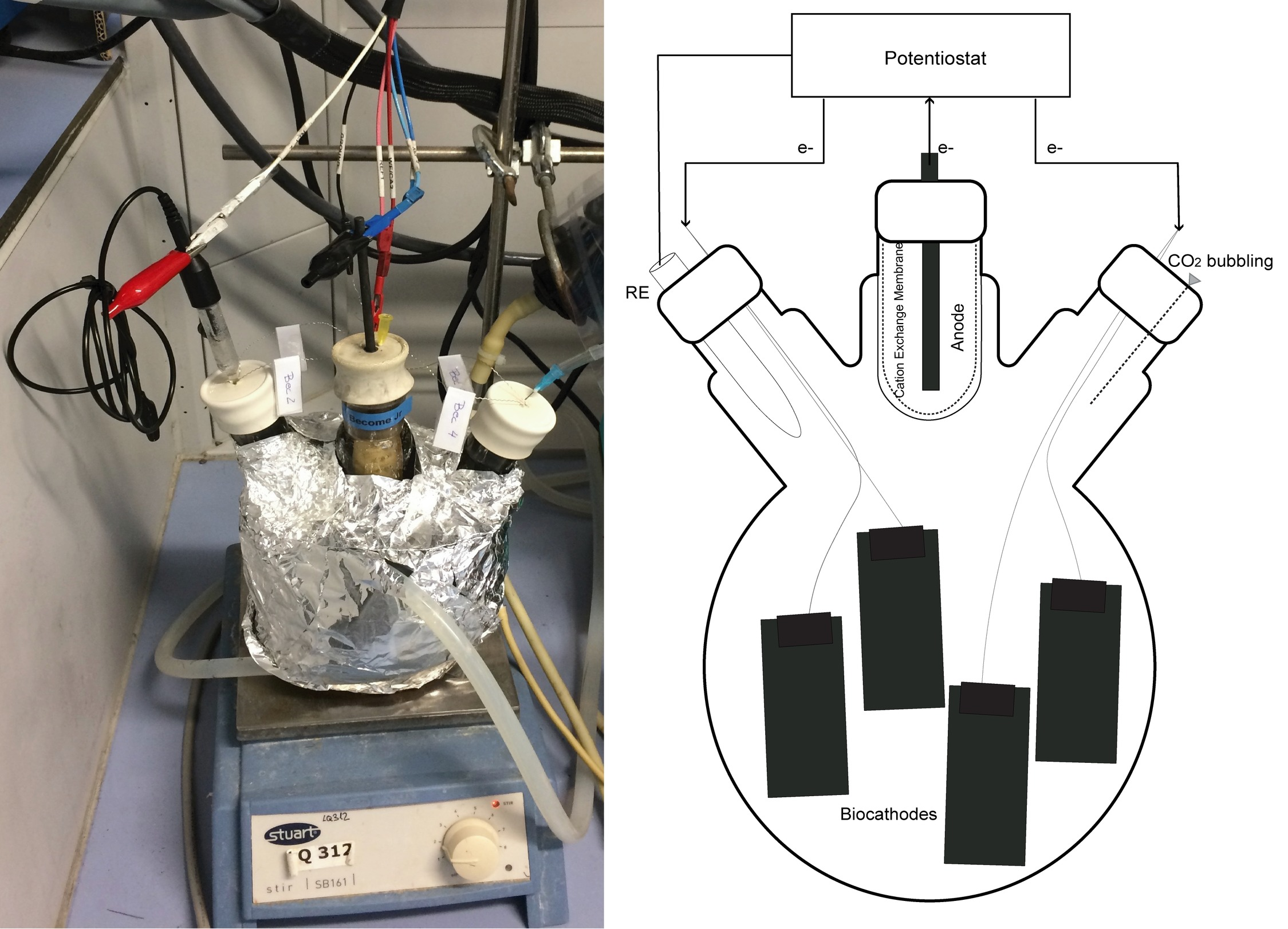

Supplement: S1 Fig — (TIF) [file pone.0215029.s002.tif]

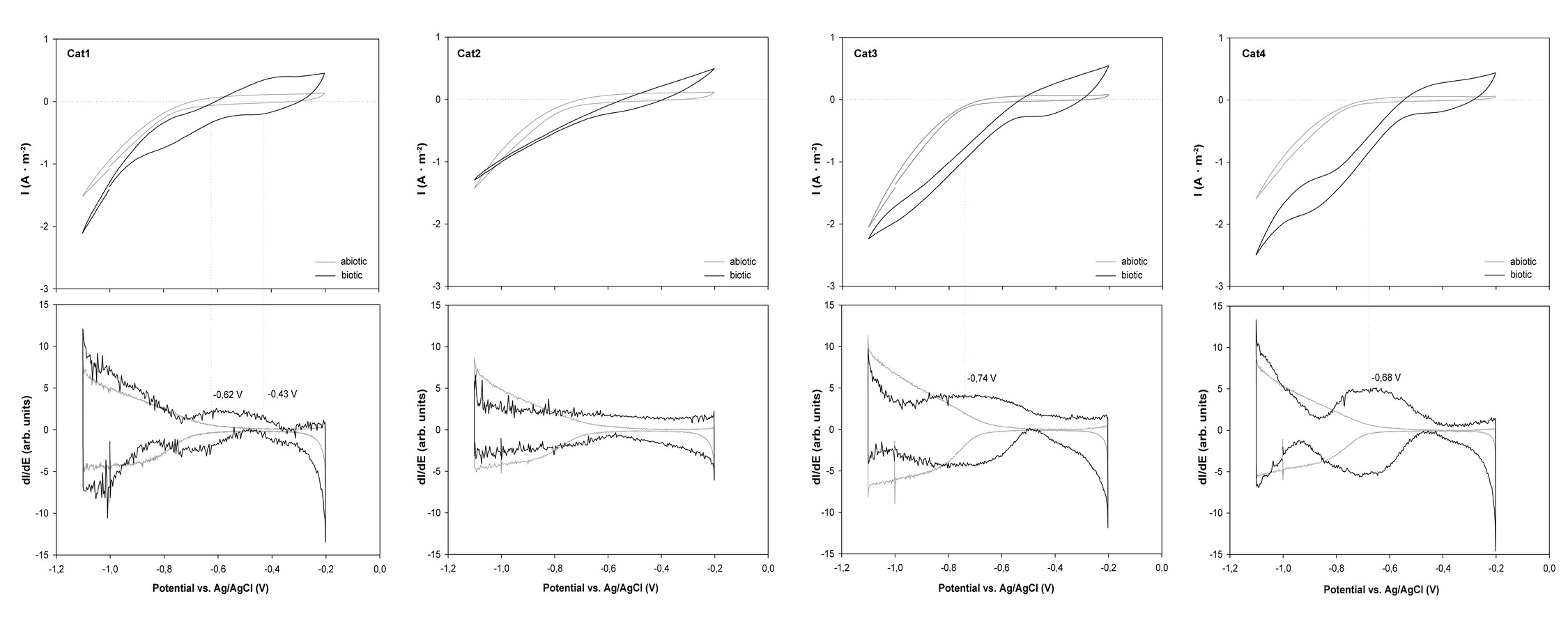

Supplement: S2 Fig — Cyclic voltammetry (CV) tests for each electrode (above) and first derivative of the respective CVs (below) under abiotic (grey) and biotic (black) conditions. (TIF) [file pone.0215029.s003.tif]

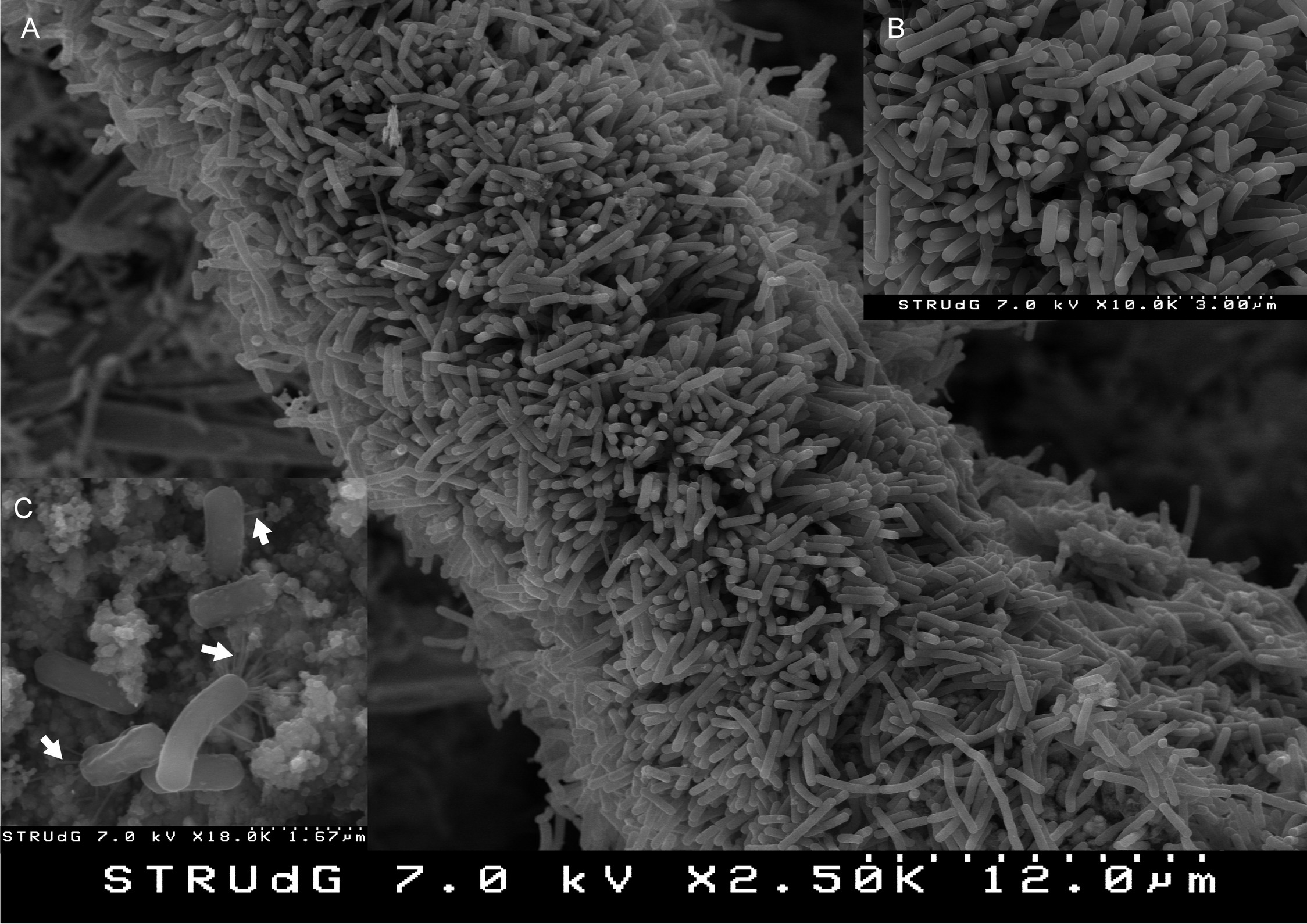

Supplement: S3 Fig — (A) Colonized carbon cloth fiber. (B) Detail of rod shaped microbes. (C) Thin appendage-like structures (white arrows) between microorganisms and carbon cloth surface were observed. (TIF) [file pone.0215029.s005.tif]

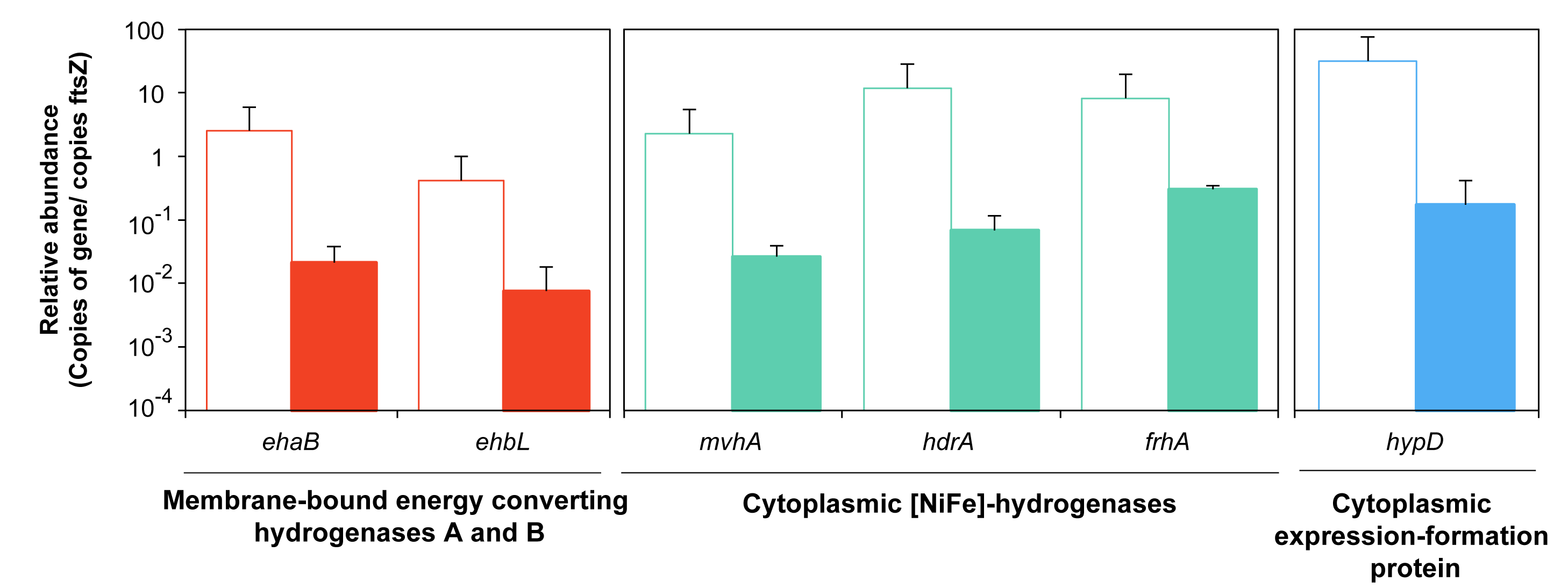

Supplement: S4 Fig — Open (empty bars) and closed (filled bars) electric circuit conditions were analyzed. Mean values and standard deviations are represented in the bar chart. Relative abundance is expressed using logarithmic scale. ehaB–energy-converting hydrogenase A subunit B. ehbL–energy-converting hydrogenase B subunit L. mvhA—heterodisulfide reductase associated [NiFe]-hydrogenase subunit A. hdrA—heterodisulfide reductase subunit A. frhA—Coenzyme F420-reducing [NiFe]-hydrogenase subunit A. hypD–hydrogenase formation protein hypD. (TIF) [file pone.0215029.s006.tif]
