# Supplementary material for: [NiFe]-hydrogenases are constitutively expressed in an enriched Methanobacterium sp. population during electromethanogenesis
Source: PLoS One. 2019 Apr 11;14(4):e0215029. doi: 10.1371/journal.pone.0215029 (PMC6459506; doi:10.1371/journal.pone.0215029)
Supplement: S1 File — (DOCX) [file pone.0215029.s004.docx]

Supplementary Material and Methods

**[NiFe]-hydrogenases are consistently expressed in an enriched *Methanobacterium* sp. population during electromethanogenesis**

Elisabet Perona-Vico^1*^, Ramiro Blasco-Gómez^2^ , Jesús Colprim^2^, Sebastià Puig^2^, Lluis Bañeras^1*^

^1^ Molecular Microbial Ecology Group, Institute of Aquatic Ecology, University of Girona, Girona, Spain.

^2^ LEQUiA, Institute of the Environment, University of Girona, Girona, Spain.

*Corresponding authors

E-mail: [lluis.bañeras@udg.edu](mailto:lluis.bañeras@udg.edu) (LB)

E-mail: [elisabet.perona@udg.edu](mailto:elisabet.perona@udg.edu) (EPV)

**Scanning electron microscopy (SEM)**

Samples were immersed in a 0.1 M cacodylate buffer solution at pH 7.4 with 2.5% (w/v) glutaraldehyde for 4 hours. After immersion, they were washed twice with cacodylate buffer and water, and dehydrated in an ethanol series. Dehydration with graded ethanol followed temperature steps of 50, 75, 80, 90, 95 and 3 times of 100 ºC in periods of 20 min. The fixed samples were dried with a critical point dryer (model K-850 CPD, Emitech, Germany) and sputtered-coated with a 40 nm gold layer. The coated samples were examined with a SEM (model DSM-960; Zeiss, Germany) at 20 kV. Images were captured digitally by ESPRIT 1.9 BRUKER software (AXS Micro-analysis GmbH, Germany). All analyses were performed in the Serveis Tècnics de Recerca (STR) at the University of Girona ([www.udg.edu/str](http://www.udg.edu/str)).
